# Breaking the silence on gendered harassment and assault of community health workers: an analysis of ethnographic studies

**DOI:** 10.1136/bmjgh-2023-011749

**Published:** 2023-05-19

**Authors:** Svea Closser, Marium Sultan, Roosa Tikkanen, Shalini Singh, Arman Majidulla, Kenneth Maes, Sue Gerber, Anat Rosenthal, Daniel Palazuelos, Yihenew Tesfaye, Erin Finley, Roza Abesha, Ann Keeling, Judith Justice

**Affiliations:** 1 International Health, Johns Hopkins Bloomberg School of Public Health, Baltimore, Maryland, USA; 2 Sociology and Political Science, Norwegian University of Science and Technology, Trondheim, Trøndelag, Norway; 3 Anthropology, Oregon State University, Corvallis, Oregon, USA; 4 Independent Consultant, Truchas, New Mexico, USA; 5 Health Systems Management, Ben-Gurion University of the Negev, Beer-Sheva, Southern, Israel; 6 Blavatnik Institute of Global Health and Social Medicine, Harvard University, Cambridge, Massachusetts, USA; 7 Social Anthropology, University of Gondar, Gondar, Ethiopia; 8 Psychiatry and Behavioral Sciences, UT Health San Antonio, San Antonio, Texas, USA; 9 Independent Consultant, Gondar, Amhara, Ethiopia; 10 Women in Global Health, Geneva, Switzerland; 11 Institute for Health and Aging, University of California at San Francisco, Berkeley, California, USA

**Keywords:** Public Health, Health services research, Health systems, Qualitative study

## Abstract

**Introduction:**

Across a variety of settings, women in tenuous financial circumstances are drawn to community health work as a way to advance themselves in the context of limited employment options. Female Community Health Workers (CHWs) are often preferred because they can more easily access mothers and children; at the same time, gender norms are at the heart of many of the challenges and inequities that these workers encounter. Here, we explore how these gender roles and a lack of formal worker protections leave CHWs vulnerable to violence and sexual harassment, common occurrences that are frequently downplayed or silenced.

**Methods:**

We are a group of researchers who work on CHW programmes in a variety of contexts globally. The examples here are drawn from our ethnographic research (participant observation and in-depth interviews).

**Results:**

CHW work creates job opportunities for women in contexts where such opportunities are extremely rare. These jobs can be a lifeline for women with few other options. Yet the threat of violence can be very real: women may face violence from the community, and some experience harassment from supervisors within health programmes.

**Conclusion:**

Taking gendered harassment and violence seriously in CHW programmes is critical for research and practice. Fulfilling CHWs’ vision of health programmes that value them, support them and give them opportunities may be a way for CHW programmes to lead the way in gender-transformative labour practices.

**WHAT IS ALREADY KNOWN ON THIS TOPIC**

**WHAT THIS STUDY ADDS**

- This paper provides a new exploration of the dynamics around CHW violence across contexts and provides suggestions for ways forward generated by CHWs themselves.

**HOW THIS STUDY MIGHT AFFECT RESEARCH, PRACTICE OR POLICY**

- Taking gender-based violence and sexual harassment seriously in the design and ongoing implementation of CHW programmes is critically important.

##  Introduction

An eerie calm falls in Sara’s urban neighbourhood, as the whirring of pedestal fans and plug-in radios abruptly ceases in the early morning hours—like many public services, electricity is a luxury in these parts of her city. (To protect participant identities, all names in this article are pseudonyms.) The heat penetrates through Sara’s barred rusty window and into her sparsely adorned single room home where her husband and three children sleep. Sara gets up, prepares food for her family, and readies to leave for a day of community health work. As she leaves the house, she lowers her head and walks at a brisk pace, moving quickly past areas where she has previously endured verbal taunts from groups of men.

After stopping by the community health centre, Sara heads towards the first household on her list; today, she is working on a door-to-door vaccination campaign. Like most other Community Health Workers (CHWs) in her city, Sara chooses to travel on foot; using public transportation would cut too far into her badly needed paycheck. Reaching a populated street, she goes down a thin alley, where she will begin her work for the day. It is in a part of the city known for ongoing violence. Another CHW was shot at in this same alley 3 months ago, but the bullets had missed her. Sara moves quickly.

Sara’s body stiffens at the sight of one door—the man inside said he would kill her if she came back. She skips it and approaches the adjacent house. A shirtless man, hair messy from just having rolled out of bed, opens the door and steps to within inches of her, telling her to come inside. Sara asks him politely if there are any women at home she could talk to. The man’s eyes dart up and down the street. His children are sleeping, he says; Sara should step inside to vaccinate them. The hairs on the back of her neck stand up—something does not feel right. She excuses herself and says she’ll come back later when the family is awake. The man curses at her. Sara tells him again, politely, that she will come back later and walks away, fearful he will follow.

At the end of a long day, Sara returns to the refuge of a communal space in the health centre. On this day, however, Sara deadens her eyes and lowers her gaze as she passes her supervisor snarling at a teary-eyed colleague, threatening to fire her for an unwashed dish.

### Violence, gender and CHWs

In Sara’s city, as in many cities across the world, public spaces (such as streets and parks) are predominantly occupied by men, whereas a large proportion of the spaces within the home are gendered female. Many women do both paid work (eg, piecework, teaching) and unpaid labour (childcare, cooking) from within the domestic space.

CHWs globally are overwhelmingly female.^1 2^ This is partially because accessing spaces inside households is often difficult for men, and because across a variety of programmes and contexts, women in desperate financial circumstances are drawn to working as a CHW as a potential way to make money in the context of limited available options for paid labour.^3^,^10^ Current estimates are that 70% of the global CHW workforce is women, but only 14% are paid adequately.^11^ Female CHWs often have few opportunities for advancement, as although women make up a large portion of the frontline workers globally, men tend to dominate at higher levels.^6^ In societies where women do not have the same freedom of mobility as men, restricted mobility may be used as a justification to exclude them from managerial roles.^7 12 13^

Gender norms are at the heart of many of the challenges and inequities that female CHWs encounter. Breaking social norms around mobility and work outside the home can lead to community backlash—where women are judged to be immoral or not respectable—particularly if they must travel without a male family member in public.^3^ The need to gain familial support is a common theme in many accounts of women seeking employment within community health programmes.^6^ A lack of social authority in public spaces increases the risk of harassment, abuse and violence in those spaces. Despite this, in many contexts, women value the work that they do and see it as morally correct. Serving their community, increasing access to health services and gaining knowledge to care for their own children allows women to harness and own the gendered role of ‘carer’.^14 15^

Yet CHWs inhabiting such gendered ‘caring’ roles are often perceived as inferior to those in curative roles, limiting their recognition within institutional hierarchies.^16^ Gender also plays out in other ways in the workplace, especially within the domains of renumeration, recognition and job mobility.^6^

Sexual harassment within the workforce is likely common but largely absent from the literature on CHWs. The gang rape of a CHW, an Anganwadi Worker, in India in 1992 led to national sexual harassment laws in that country, but sexual harassment and violence from supervisors and community members persist.^17^ In a survey of female CHWs in Northern Karnataka, India, Rao *et al* found that 32% experienced sexual violence and 26% experienced physical violence; this violence happened at home as well as in the workplace, including violence perpetrated by members of the families they served.^18^ Mumtaz *et al* found that female CHWs in Pakistan face regular harassment and abuse from seniors and coworkers, in addition to hostility from the community and their own families.^4^ While most of the evidence in the literature is from South Asia, this is an issue with global reach; other articles have documented rape and fear of rape by CHWs in Kenya and the DRC.^6 19^ Sexual harassment from community members and supervisors has been documented in a range of contexts.^4 20 21^ Then, as the examples of COVID-19 and Ebola illustrate, violence against CHWs is often heightened during health emergencies.^22 23^

Yet for the most part, the literature is silent on the pervasive issue of violence against CHWs. Most articles that include information on violence or harassment mention it as a secondary finding. Part of the reason for this is that making these issues public can put vulnerable workers at risk of retaliation. Here, we, a group of researchers and implementors who engage CHW programmes across the world, aim to break the silence on these issues by providing details of the gendered harassment and violence that CHWs face decoupled from the identifying details that could endanger these CHWs. Our goal is to shed light on a critically important issue.

Here, we present evidence from three studies—two discussed briefly, one explored in depth—regarding harassment and assault in CHW programmes. We also provide suggestions from CHWs themselves for making CHW work safer. We conclude with a framework for understanding the determinants of CHW harassment and violence as well as some thoughts on the way forward, both in research and practice.

The CHWs we work with have a vision of transformative health programmes—ones where violence and harassment are not ongoing problems. At the centre of this vision are female CHWs who are valued, supported and empowered to participate in creating opportunities for advancement, both personally and for CHWs generally.

In the institutional context of frontline global health labour, this vision is radical. The CHWs we talked to believe it is possible.

## Methods

Because the topic of gender-based violence is so sensitive, we felt we could not use specific examples from identified research projects without putting our research participants at risk. Thus, we are not providing identifying details of the studies we are drawing from here. These studies took place in different countries, explored different types of programmes (government organised and organised by international organisations) and were conducted over a range of decades.

Example 1 is drawn from an ethnographic study of an all-female cadre of CHWs within a government primary healthcare programme. That study was conducted in a remote, rural area of a very low-income country in the early days of PHC programmes in the late 1970s and early 1980s. The researcher in this study visited 24 different health posts; many of these health posts could only be reached after several days of walking. At each health post, the researcher spent a number of days accompanying CHWs on their home visits. She took detailed fieldnotes about this participant observation by hand, and later typed them up on a typewriter. The researcher also interviewed CHWs at these health posts as well as their supervisors at district and regional levels. The researcher kept all typed notes from both participant observation and interviews, enabling us to use this material as well as published material from that study in preparing example 1. Example 1 illustrates that the issues we discuss in this paper are not new but have been part of CHW programmes for decades.

Example 2 is drawn from a study of CHW experiences in another all-female government-organised programme, in a different country, several decades later. This study was commissioned by an international donor in order to better understand the experiences of CHWs in the country. The researcher interviewed 36 CHWs and 33 CHW supervisors working in a number of different settings across the country. These interviews were audio recorded, transcribed and translated. The researcher took notes about the context and setting of the interviews she conducted. We used interview transcripts and notes from this study in preparing example 2.

Example 3 is drawn from a recent study of an internationally organised and funded CHW programme, implemented in collaboration with local governments and carried out by a local research team with a deep understanding of cultural context. (The members of that research team have asked to be left off of the author list and acknowledgements of this article in order to protect research participants.) That study, carried out over a period of nearly 2 years, included a number of components. First, it included more than 100 interviews with CHWs and their supervisors; these interviews were recorded, transcribed, translated and coded for major themes. For this paper, we drew on codes relating to gender, harassment, community interactions, violence, motivation and hierarchy. This study also included participant observation in CHW work; a team of four researchers followed CHWs and their immediate supervisors in their tasks for a month, taking detailed fieldnotes. Researchers spent time joining workers in their work, eating with them and becoming familiar with the structure of their days. Finally, the study included sessions where groups of CHWs discussed their work and documented their concerns and thoughts. We drew on all three of these bodies of evidence—interview transcripts, detailed fieldnotes from participant observation and documentation produced by groups of CHWs—in example 3. The more serious concerns presented in this paper were documented after a 2-year process of relationship building. Sara, the woman whose story begins this paper, was a participant in this study, and those experiences were documented during participant observation.

### Ethical review

Study protocols for example 2 and example 3 were reviewed and approved by the relevant Institutional Review Boards (IRB) at the time of the research. The research presented in example 1 was conducted before IRB review was required for ethnographic studies, but the Johns Hopkins Bloomberg School of Public Health IRB has reviewed and approved our use of material from that study.

### Patient and public involvement

Patients were not included in the studies presented in example 2 and example 3; patients were included in the study presented in example 1, but we did not use that material here. In all of our examples, we prioritised the perspectives of CHWs, as they are naturally the people who have the most expertise on the issues of CHW harassment, violence and safety.

The funding source for these studies had no role in this paper; we have provided them with the manuscript and have received no feedback. We have also provided these results to other stakeholders in these projects.

### An ethnographic approach

Here, we present information from examples 1 and 2 briefly, and then spend the bulk of the paper exploring example 3 in some depth, using a specific case to flesh out the dynamics at play. We also pull our observations into a framework to facilitate use of this material in policy and practice. We constructed this framework inductively, using the material from our three examples. As we are drawing on ethnographic studies, we follow ethnographic conventions in this paper, aiming to present as much texture, narrative and context to what we are describing as possible, while still maintaining the confidentiality of our participants.

## Results

### Example 1

Two women, one aged 17 and one aged 18, stood nervously and alone outside a rural health post, keeping their distance from the male staff of the post. It was the early 1980s, and these women were part of a new cadre of community-based workers for maternal health.

These women were stationed far from home and family, so they had to find housing on their own in this unfamiliar community. They were the only women stationed to work at these newly created health posts.

Women working for this programme were scared. They spoke in interviews about how difficult the work was, and how alone they felt. They commented that the health post supervisor expected them to come to his house and act as his personal maids. They also said that they were frightened of sexual advances from coworkers.

Supervisors appreciated that things could be hard for these women at first but commented that the women tended to adjust with time. The discourse among policymakers in the capital was about how important it was to develop careers for women, which donors could point to as evidence of gender empowerment following 1975’s International Woman’s Year.

Little attention was given, in any discourse around this issue, to the possibility that women might be scared because there were very real threats of violence, sexual assault, and harassment, or that women might be frightened because they had already experienced these things.

### Example 2

On a hot afternoon in the middle of summer in a densely populated urban area, a CHW took a break from her ongoing work to sit with an interviewer in an empty room in a health post. The two of them sat in the quiet room on mended chairs next to a broken freezer, discussing CHW work. In the middle of the interview, responding to a question regarding support from her supervisor, tears welled up in the CHWs’ eyes:

I don’t understand what my supervisors think of me. It’s really, it’s wrong. The way they look at me and what I do. They will say *anything* to me. Some people, they don’t have the respect that they should have…

A human deserves respect, whether they’re a man or a woman—really, you’re supposed to give more respect to a woman. But I don’t feel any such respect here.

Look, I just try to get my work done.

Here, the interviewer, a coauthor on this paper, chose not to probe and changed the subject.

Later, a male supervisor at a neighbouring health post, in response to a general question about supervision, said that CHWs were ‘afraid of punishment and assault’—both by their supervisors and in the communities they served.

### Example 3

Sara’s CHW programme was a particularly challenging one: she worked in a violent context, where community members did not trust the government and the international organisations she represented. The challenges she and her colleagues encountered throw issues of gender and violence into relief; many of the challenges common to CHW programmes across settings were present in extreme forms in this context. We are spending the rest of the results section of this paper exploring the challenges Sara and her colleagues faced in depth as well as the solutions that CHWs suggested to solve these problems. We think this example highlights the complexity of this problem and the urgent need for action.

In this programme, respondents at all levels of management agreed that women were best for CHW work because of their ability to access gender-segregated spaces inside the home. A supervisor commented:

It is good that women are hired for field level work, as a woman can go inside the home, perform her duty accurately, and leave no stone unturned…. A woman can provide more accurate data than a man, as he cannot go inside.

Many CHWs in this setting chose to do the work because they desperately needed income and because there were few other employment options available. Living-wage employment was hard to find for nearly everyone living in the centre of this city—men and women alike—but the shortage of jobs was particularly acute for women. CHW work for this programme paid slightly above minimum wage, and supervisors received stacks of resumes for open CHW positions. One CHW told us:

After the death of my husband I was looking for a job desperately… I have a daughter and I need to do something for survival. Thank God, I got this job and am working in this program.

Many women had applied for CHW positions due to the death or disability of a male family member. Still, they frequently faced opposition from family members over their choice to take the job—many families were reasonably concerned about women’s personal safety in doing door-to-door work. A CHW explained:

I have three children and I didn’t have sufficient resources, so I thought of getting a job. I was working in a house [as domestic staff] and getting [$12 USD] per month. A CHW regularly visited that house, she told me about this job, and I applied… I got the job, but my husband was not allowing me to join, as he was afraid [for me]… We discussed it and he eventually allowed me. I am thankful to God I got this job. Though I receive a low salary… it can be used for our children’s education.

Although many women took the job out of financial desperation, once they were in the position, they felt proud to serve their communities and felt that they made a difference. One CHW said her motivations had changed over time:

I had little choice but to take the position, and the salary was good. Before joining this job, I didn’t know much about it, but later I was happy that we provided a service and created awareness.

That this work was well paying, in a space with so few other options, meant that women felt they could not leave their jobs. The job carried a range of acute risks, both physical and social, but the value of a job that paid above minimum wage was sufficiently enormous—and rare—for women in this context that it was worth it to them to weather those risks.

#### Gender role policing by the community

In this context, where local norms restricted women’s mobility, and where there was community mistrust towards government and international actors, going door-to-door carried the potential to damage women’s reputations. This was an enormously materially important matter for CHWs in this city; gossip could lead both to community rejection and the loss of family support for working outside the home.

CHWs were constantly aware of how their actions appeared to others. They endeavoured to be polite and open, but not too friendly; well-kept, but not trying to attract male attention. They tried not to seem too casual with their male supervisors, while still maintaining good working relationships. They tried to maintain a delicate balance.

Yet they still faced censure. ‘Even in the community where I live’, one supervisor told us, ‘they say that the women who work as CHWs are not morally upright’. A CHW commented:

Sometimes a male supervisor goes with us into the community and people say, “Look at them—they are roaming with a man!”

And a female supervisor explained:

We hear these sorts of comments: “Why don’t you stay at home?” “Why don’t you take care of the children?” “Why don’t you work at home? You can do many things at home.” “You only like to wander in the streets.”

#### Violence from the community

Aside from pervasive anxiety about presenting themselves in a respectable manner, CHWs (and their male managers) also faced severe, intermittent violence in the course of their work. This ranged from being yelled at, to being hit with household objects, to being threatened with murder. A supervisor described physical violence against CHWs:

Like once a lady spilled water on a frontline worker, and at another house an old man was furious with a frontline worker and… threatened to hit the worker with a shoe. In the same area, a female supervisor was hit with a heavy pot.

Anxieties about personal safety followed workers home, as most CHWs worked in the areas where they lived. The relationships they developed with the community mattered not only on duty but also on off as well.

Female CHWs were particularly vulnerable, due to their gender, their social class and the economic circumstances that lead them to seek the work in the first place. Those in the community understood their vulnerable situation and often felt empowered to treat them badly because they knew there were unlikely to be consequences. We heard many stories of verbal and physical violence against workers happening with impunity. A CHW described the issue:

The problem is born when parents believe that if they disrespect, misbehave, or assault [CHWs], nothing will happen.

In cases where community members hit CHWs and even drew weapons, it was usually the case that no action was ever taken. CHWs feared registering reports with the police because they did not want to make enemies in the communities they lived and worked in. Also, we heard many stories of CHW staff going to the police to file complaints only to be told that their complaint of harassment or violence was a ‘personal matter’. The fact that these stories circulated among CHWs reflect a widespread feeling among these cadres that they had little recourse in such cases.

Some CHWs told us they felt that the programme that employed them had a duty to support them when such incidents occurred, as the violence they faced was related to their role in the programme. Among other backing, they wanted legal support to prosecute cases. A group of CHWs explained:

This fight or unhappy incident happened because of the program, they hit us because of the program, this is not a personal matter that we should deal with ourselves. Fights come all the way to our house, and we make enemies. Then our family pressures us to leave the job.

Another CHW said:

The first responsibility of any organization, whether private or public, is to take care of its workers.

Yet this, too, was complicated. Local leadership said they would be happy to support workers legally if they were to take a case to court. However, they commented, few workers could socially afford to lodge a case against someone in the community because this could start a feud between families. These issues went far beyond the health sector into local social dynamics, and all this led to a pervasive sense among workers that regardless of what they did—talking to supervisors, going to the police station, registering a complaint—action would not be taken.

#### Workplace hierarchy

These anxieties reflected another underlying dynamic: if relationships with the community were complicated, relationships in the workplace were also complicated. While CHWs were women, many managerial positions were filled by men.

Women and men alike agreed that at least some men were needed in managerial positions because of men’s ability to travel, their access to men in positions of power and influence in the community and their access to male-dominated spaces. A female area supervisor echoed the sentiments of many of her colleagues when she commented that ‘there are many things which are more easily handled by men’, citing visits to schools, local governance structures and local religious leaders as examples. Many respondents also noted that while there were real advantages to male managers in their ability to access male spaces, more gender diversity in managerial positions would nonetheless be welcome.

Workplace dynamics played out differently in various health centres. In some, managers were supportive, creating what one worker called a ‘family-like’ atmosphere. In others, CHWs were not empowered to take breaks or express their opinions, they were yelled at often for small mistakes, talked down to and made to do menial tasks outside their job descriptions. A team of CHWs said:

Our seniors threaten us with being fired, they do whatever they like, and they disrespect us in whatever way they want. They take our mistake and make it a big issue, and disrespect us in front of everyone. If they explained things to us nicely then we would understand and not make the mistake again.

In some but not all health centres, we observed sharp divisions between levels of workers, with the highest salaried workers getting the most respect. These issues were gendered, since CHWs were mostly women and their managers were mostly men, but they were not only about gender. Female supervisors took advantage of hierarchies too. Some CHWs said of their female immediate supervisor:

For example, if we want to go out we need the supervisor’s permission, if she allows it, then we can go. Even for going to the bathroom we need permission. We cannot do anything without permission of the supervisor. We are treated like children. We have been asked to clean this place [the health center]…They yell at us asking why this place is not cleaned, why the utensils are not cleaned.

We also observed health centres with more equal relations between staff, where the team all sat in one room having open and polite discussion about the updates, questions and concerns of the day. In some health centres, supervisors and managers spoke respectfully, even to the youngest and newest CHWs, who are most vulnerable. What this indicates is that unhealthy hierarchies are not an inevitability in this city, and that the knowledge and capacity to do things differently exist in local structures. The fact that different leaders set different tones for their staff suggests that that it would be possible to implement effective strategies that provide improved leadership while protecting workers.

#### Sexual harassment

A particularly painful aspect of workplace hierarchies and the vulnerability of CHWs in the setting of example 3 was sexual harassment. CHWs told us that many workers simply remained silent about harassment, especially as being open about their allegations could harm the reputation of female staff. One CHW had tears in her eyes as she told us she wanted to throw something in her supervisor’s face and leave her job, but that she could not because she needed her job to survive.

A group of CHWs commented:

The issue is, most of the staff are female and alongside that are victims of harassment. They stay in a state of fear, and this leads to their not being able to do their work properly; so the program is affected. They cannot share this [the harassment] because they want to protect their honor.

Concerns about protecting honour were very real. A harassment allegation could boomerang into a character smear with severe social and economic consequences. This made it difficult for CHWs to come forward.

Often, CHWs sought out female managers to discuss these issues. However, these female managers were also relatively low in the administrative hierarchy, and passing these comments up the chain to their male supervisors was not always effective. Some sent anonymous letters to the district-level office.

As with other types of harassment, sexual harassment was a problem limited to certain areas; it could be acute in those areas, and it was absent in others. We observed, and female researchers on our project experienced, comfortable and supportive relations in many health centres. A female low-level supervisor from one of those health centres said:

We feel comfortable working with men. We work like a family; we respect each other and do not feel apprehensive [working together].

But in health centres where harassment was an issue, CHWs told us, harassers felt that consequences were unlikely. A group of CHWs explained:

The biggest reason for this issue is that there is no harassment policy in the program that the staff will be afraid of, nor is there a reporting line that the lower-level staff can access. Additionally, the officer who commits the harassment is the reporting line himself! So, the lower-level staff stays quiet, and the harassment continues.

There was in fact a harassment policy on paper, but workers did not know about it, nor did they have support in navigating systems were they to report. The anxiety about reporting coalesced into anger for some. Another group of CHWs commented:

We workers have some issues at the health center that we can't discuss with our seniors. Whatever issues workers are confronting, there is no way to express our problems and no one to listen to us. There is no one to take our problems. CHWs are given no honor. No one gives importance to what workers say. Workers are disrespected, and workers have no self-respect.

Officially sexual harassers were supposed to be fired; in practice, outcomes varied. Sometimes perpetrators were transferred—solving the problem in one area but reproducing it in another. Occasionally, action against perpetrators was taken, as in the case of one supervisor who was removed after the third case against him. Although there have been ways to report harassment and official mechanisms for dealing with complaints, what we heard from many workers was that they either did not know about these or felt they could not safely report what they were facing at work.

The health system in question is currently working to address this issue, with an increased focus specifically on harassment. There are dedicated officials to address this issue in the organisation that contracts CHWs, and a reporting hotline has been opened. These efforts have started showing results in terms of bringing issues to the fore, investigating them and taking concerted action including warning letters, transfers and job termination for harassers. Yet the issues around fear of victim blaming persist; building trust around these structures will take time.

#### Gender, power and coverage of health interventions

CHWs in the example 3 study outlined a number of ways that the dynamics above interfered with their ability to provide quality health services. First, when workers were scared for their safety in the field, they could not focus on activities like social mobilisation and data collection in the relaxed and meticulous way necessary for good relations and high coverage of interventions. A group of CHWs explained the downward spiral they experienced:

Anyone can get up and curse at us. In the street, any woman who is refusing a vaccine can stand up and curse us out in front of everyone, and all the male shopkeepers make fun of us. Because of this, we feel badly about ourselves. We feel insecure and cannot focus on our work. This negatively affects the program, and we become mentally disturbed. This further negatively affects our performance, and we make mistakes in the data collection. When there is a mistake in data, then workers are talked down to, but no one focuses on our stress and insecurity. Because of this, the program suffers. Because of this, [our country] suffers.

Another group of CHWs said:

Workers are disrespected both in the community and at the health center… Because of these stresses we cannot work as we should. We cannot present the program to the community the way we should.

Second, workers commented that when community abuse went unchecked, it exacerbated community perceptions that they had no real government backing, and their work was thus taken less seriously. They told us that being backed up in cases of community conflict was important not only to their well-being but also to their legitimacy in the community. A team of CHWs explained:

If the government gives us full protection, then we will work with great determination, the people will support us, and coverage will be high. The community will also know this is not a frivolous program, the government is backing us.

Many CHWs commented on the connection between respect for workers, the ability of these workers to do their job properly and the success of the programme:

The institution who cannot take care of its workers loses respect in the community. In this type of institution no one can do their work well, and the institution cannot fulfill its goals.

#### Suggestions for change

CHWs in example 3 argued that programmes should continue to employ vulnerable women in low-income communities: they are an extraordinary asset, and they value their employment enormously. They further argued that these programmes should be aware that employment can increase the risk of violence for these women, and should take it on themselves, as a basic HR measure, to plan for ways to prevent and mitigate that violence. CHWs suggested a number of steps that could be taken to protect them in their work.

First, to reduce community harassment, CHW teams called for increased legitimacy and support from the government. They suggested documentation such as ID cards, and public statements delivered via advertising and news programmes, affirming that they are valued government workers.

CHWs were calling for legitimacy, one of the building blocks of trust. CHWs clearly described the connection between their employer taking public responsibility for their well-being and the level of legitimacy they enjoyed in the community. Their gender made legitimacy an acute issue, as CHWs were already going against norms of respectability by working door to door.

To tackle harassment by supervisors, CHWs suggested establishing a system where complaints could be made anonymously and carefully investigated. Multiple groups of CHWs called for more external monitoring, increased space to air grievances and one-on-one sessions with those in charge. One group of CHWs suggested holding regular harassment trainings, perhaps even every 2 months, to aid in culture change. Additionally, they suggested that candidate screening during the hiring process for supervisors should be improved.

Apart from protection against violence and harassment, CHWs wanted to have the space and support for job advancement. One specific idea was for CHWs to be allowed time off to take the examinations to gain the qualifications needed for higher level positions. Multiple CHWs pointed out that being passed over for management-level positions was demoralising, and that experience within the programme should be considered when hiring for these roles. CHWs wanted hiring for management roles to be based as much on performance as qualifications, which would ultimately result in more women from varied backgrounds in leadership.

The programme described in example 3 is in many ways an extreme case; CHW work placed women in this insecure context at additional risk of violence. The issues here are thrown into sharp relief, but they are not unique. As Asha George and colleague have argued, violence against female health workers is the ‘tip of the iceberg’ when it comes to the gendered issues that female health staff face.^16^ Examining the dynamics surrounding violence can give insight into how to best support female CHWs in less extreme circumstances as well.

### A framework for understanding CHW harassment and violence

**Figure 1:**
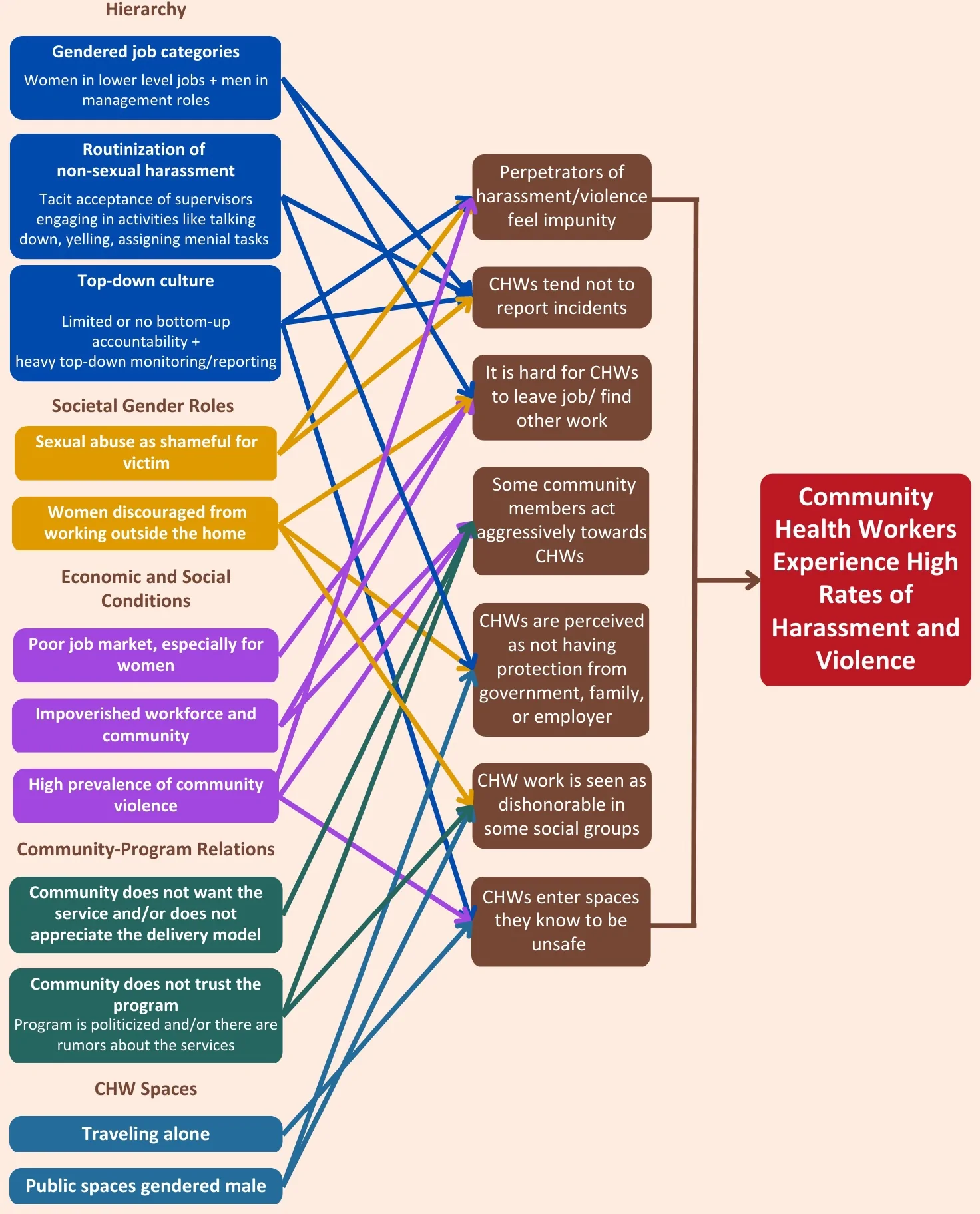
Framework of determinants of CHW harassment and violence. CHW, Community Health Worker.

These three examples make clear that there are particular aspects of programmes—and the societies in which they are embedded—that make CHW harassment and violence more likely to happen, less likely to be reported or both. The framework in figure 1 draws outlines these determinants on the left and shows their effects. Identifying the determinants of violence is useful because it provides specific arenas in which to intervene to reduce the likelihood of these events.

## Discussion

Violence against female CHWs is an issue with global reach.^16^ These issues are often not talked about openly: doing so can cause reputational risk to health programmes, and it can be politically easier to look aside, but airing them and addressing them is key both to supporting female staff, and to achieving real progress on health programmes aimed at the people they are best positioned to reach.

As our framework suggests, certain structural characteristics of CHW programmes put CHWs at high risk. As the International Labor Organization (ILO) argues, ‘violence does not reside only in individuals’ intentional acts but also in certain organisational practices and characteristics’.^24^ These practices and characteristics can be identified and altered to create safer work.

Across the world, from global movements like #metoo to local movements like the Aurat Marches in Pakistan, momentum is building for change. It is time to put these issues much closer to the centre of the action and research agendas.

### An action agenda

Some of the domains on our framework—societal gender roles, and economic and social conditions—are largely beyond the power of health programme administrators to change. But others—hierarchy, community–programme relations and CHW spaces—are within the purview of health policymakers. Here, we review action steps for each of these areas that underlie CHW vulnerability. We also review best practices for dealing with harassment and violence when it occurs.

The specific vulnerabilities and structures affecting CHWs are different in different settings, and models tailored to context are needed. Still, throughout these recommendations for action, there is a common thread: *workplace harassment and violence is connected to other forms of power*.^25^ Creating more egalitarian health systems, ones where the voices of CHWs are heard and respected, is the key ingredient in reducing their vulnerability.

#### Hierarchy

Three domains of our framework relate to hierarchy on the job. The research projects show that when women are in low-ranking job categories with men in management roles, when non-sexual harassment is routine and tolerated and when there are heavy top-down mandates and accountability, the foundation is laid for high rates of sexual harassment and violence. Scholars of workplace violence, as well as the ILO, agree that hierarchy is a key ingredient in creating and sustaining the conditions for work-related harassment and violence.^24 26^

One clear take-away from the work featured here is that leadership matters. Good supervisors create the enabling conditions for healthy relationships, but the organisational structures that those leaders exist *within* are an even more important enabler of healthy workplace culture.

The literature identifies fostering more egalitarian workplaces as a key way to reduce vulnerability.^27^ The ILO includes the following in its list of risk factors for harassment and violence: workers lacking control over how work is done; autocratic leadership; career advancement ladders that exclude certain groups; organisations under pressure to meet difficult targets at low cost; ‘situations where large numbers of women are supervised by a small number of men;’ ‘normalisation of violence and harassment;’ ‘cultures in which bullying behaviours are not challenged’ and power dynamics that give the *perception* of impunity for perpetrators. The ILO also identifies a list of ‘indirect measures’ to reduce harassment and violence: ‘leadership, pay, management of workplace conflict, reward and recognition of effort, career opportunities, job security, working conditions, workplace consultation, communication and involvement in decision-making, control over workload, work schedules, work culture issues such as level of support, social or physical isolation and management style’.^24^

This list will sound familiar to anyone engaged with the current discussion around gender and CHWs. They align with WHO recommendations to ensure that CHWs have supportive supervison, fair remuneration, contracts that assure job stability and security and a clear career ladder.^28^ They also align with calls from WHO, Women in Global Health and others to change the situation where health programmes are ‘delivered by women, led by men’.^29^ The clear take away is this: providing more egalitarian health systems where CHWs have a voice, have the opportunity to progress and have autonomy over their work tasks improves CHW programmes, promotes gender equity *and* is likely to contribute to reducing harassment and violence.

#### Community–programme relations

Another key aspect of our framework is that harassment and violence against CHWs can be reduced if the communities they serve want the services they are providing. CHWs will also be safer if the people they are serving trust the model of service provision.

Improving community–programme relations through community participation and ownership has always been a cornerstone of primary healthcare programmes. This goal is, of course, a central piece of why CHW programmes exist in the first place. Yet responsiveness to communities is a central component of good CHW programme practice that is still sometimes overlooked.^30 31^ Our work is a critical reminder that CHW programmes must be designed to be responsive to communities, and not just policymakers. This has long been understood to be a key part of equitable, responsive, high-quality CHW programmes—what we are adding here is that it is also a key part of *safe* CHW programmes.

CHWs are often deployed to convince hesitant communities to accept health interventions in cases where there may be opposition to those interventions. They are very effective at this—but we should also be aware that this can put them at risk and pay particular attention to safety in such situations.

#### CHW spaces

A common thread across many CHW programmes that employ women is that those programmes may force women to bend or break social norms regarding gender and space in the course of their work.^32 33^ As in example 3, female CHWs are valued in such settings because they can access the female-dominated spaces of homes. But the fact that they must move through spaces gendered male to get there increases their risk of violence and harassment. As in example 1, it can sometimes also be the case that being posted in areas where they do not have social support can put CHWs at higher risk.

Awareness and acknowledgement of this fact can facilitate thoughtful design of safer systems. CHWs themselves are the experts in this arena and can suggest workflows that keep them safe. For example, safety considerations can lead to safer workplans such as events happening only during daylight hours, CHWs working in pairs rather than alone and regular reviews of CHW safety.^34 35^ Critically, CHW programmes can listen to CHWs when they report that certain homes are not safe, and not expect them to return to those homes.

#### Addressing violence and harassment when it occurs

The above measures are preventative, aimed at the determinants of harassment and violence, but when it occurs, programmes also need strategies for addressing it.

The ILO—and WHO—recommend that organisations have clear, zero tolerance policy statements to address harassment and violence in the workplace, including ‘a readiness to engage in support of any action targeted at creating a violence free environment’.^36^ Supervisors and managers must be trained and supported in this duty, and complaints should be investigated by an independent party, free from retaliation.

Some CHW programmes already include safety trainings in as part of CHW training; this should be standard practice. Training should target how to cope with violence when it happens, and also how to prevent it. Beyond training, assistance and counselling should be available to those affected by harassment and violence, and there should be regular monitoring and evaluation of the effectiveness of these structures.^36^

Such steps have been implemented in many programmes: for example, Partners in Health Malawi recently instituted a highly visible antiharassment campaign, along with clear guidelines, aimed at making work safer for all its staff, including CHWs. As another example of a model of action, a tragic serious sexual offence on a CHW in India in 2016 led to immediate directives from the national level to improve safety for CHWs in the country, including ensuring a safe place for CHWs accompanying pregnant women to health facilities at night; collecting information on harassment as part of regular meetings, and taking action based on this information; training all staff in preventing violence against women;^37^ bringing village-level accountability mechanisms to bear;^38^ creating a complaint line and providing treatment and social security for CHWs affected by violence.^39^ These measures resonate with those suggested by the CHWs in example 3.

### A research agenda

It is not only programme managers but also researchers who have neglected this issue. The studies reviewed here have several limitations in speaking to CHW violence. None of them was designed with this issue in mind. In some cases, we failed to explore the dynamics adequately. We did not include quantitative measures that would give a precise sense of the extent of the issue. Here, we suggest some ways forward.

#### Encouraging CHWs to speak

The structural position of female CHWs means that it is extraordinarily difficult for them to address these issues, and fora to discuss them openly are rare. Yet when provided those fora in a context with time for building trust, CHWs have many ideas for achieving positive change.

In the research project described in example 3, it was critical that researchers spent years building relationships with CHWs. Researchers began to be trusted with more sensitive topics over time, during informal conversations and eventually in formal documentation. In part, this is because the research findings in the early period of work lead to positive change in several areas, demonstrating the commitment and credibility of the research team.

Standard public health qualitative research approaches—one-off interviews done by strangers—are highly unlikely to get at these deeper issues. It is critical that we invest in methods like participant observation that build trust, and methods like long-term human-centred design processes that allow women to articulate for themselves how to improve the context of their work.^40^

#### Taking coded language seriously

It is also important to be attentive to the coded language used to discuss these issues. Here, for example, are some quotes where CHWs were discussing sexual harassment and assault, with the coded language bolded:

These problems come up because the supervisors tell us, “No one can say anything to us. Just pay attention to your work.” **They are not accountable to anyone for misusing their authority**. Because of this they become overconfident and apart from their duty they start to think of themselves as superior and **make problems for others**.

We workers have **some issues that we can't discuss with our supervisors**. Whatever issues workers are confronting, there is no way to express our problems and no one to listen to us… **[CHWs] are given no honor**… **Workers are disrespected both in the community and at the health post**… Because of these stresses we cannot work as we should.

To solve this problem, first of all, there should be harassment training. Every month or two months, the concept should be reinforced not to do **bad things**, and not to get involved in **immoral activities**.

As researchers, we need to be more attentive to such coded language than we have been in the past—as evidenced by the fact that no article we reviewed interpreted such language as potentially pointing to sexual harassment or assault.

#### Actually listening

On reflection, we found many examples in our own research—such as example 2—where CHWs were telling us quite clearly that something was amiss, and we chose to look the other way. Some of us, female investigators who ourselves had experienced sexual harassment at high levels of global health programmes, chose not to hear it when the most vulnerable women in these very programmes described sexual harassment to us.

Their risk was, in retrospect, obvious. But we avoided what was presented to us, and we avoided it systematically, again and again, in both the structure of our interviews and the trajectory of our writing. The issue may not be that these women were afraid to speak about these issues. The truth may be that we were afraid to hear them.

In our own writing, when CHWs’ husbands and fathers have expressed concerns about their safety in doing CHW work, we have tended to interpret that as evidence of patriarchal control. It is that in part, of course, in many cases. It may also sometimes be a very real and very rational concern for the physical safety of family members. Perhaps we should be listening better to these concerns as well.

Research can play a helpful role in moving the needle on our knowledge in this area, beginning with questions such as:

- In internationally funded health programmes, how do donors and global health agencies address harassment within their workforce, particularly their CHW workforce? Are there emerging best practices?
- Does simply having more women in higher level roles make a difference? Or do we need to also be attentive to class and ethnicity in thinking about ways forward?
- How does the amount women are paid for health worker roles change the dynamics of the job, and in what ways?
- In the face of pervasive underfunding for professionalised CHWs, what increase in funding would be necessary to institutionalise effective worker protections like Human Resources departments?

Beyond qualitative work, quantitative work can help illuminate the extent of the issue. There are many existing surveys, including the Negative Acts Questionnaire (NAQ-R), the Impact of Event Scale (IES), the Leymann Inventory of Psychological Terror (LIPT), the Inventory of Violence and Psychological Harassment (IVAPT) and the Danish Copenhagen Psychosocial Questionnaire (COPSOQ), for better understanding the extent of harassment and violence; these should be used regularly in CHW programmes.^24^

This work is not without challenges—for example, as the author list of this paper illustrates, it is difficult to uphold principles of CHW leadership and coauthorship when such leadership and coauthorship can put CHWs in danger. But these complexities are not reasons to ignore these research topics; rather, they are reasons to engage carefully and deliberately.

## Conclusion

Global health programmes, governments and powerful funders can do much more to protect CHWs from harassment and violence. There is an opportunity to implement solutions that have been effectively deployed in other sectors, and which dovetail with the CHW support and community engagement agendas more broadly.

The work of making CHW programmes safer will need to happen in hundreds or thousands of different programmes that employ CHWs. This work will not be easy, but many solutions to these problems exist. Fulfilling CHWs’ vision of health programmes that value them, support them and give them opportunities may be a way for CHW programmes to lead the way in gender-transformative labour practices. CHWs tell us that they are ready to help.

## Data Availability

Data are available upon reasonable request.
